# The Urinary Uric Acid / Cr ratio as a marker of morbidity and mortality of preterm infants: a case—control study

**DOI:** 10.1186/s12887-021-02798-7

**Published:** 2021-07-24

**Authors:** Mansour Sadeghzadeh, Parisa Khoshnevisasl, Ramezan Fallah, Asghar Marzban, Seyyedeh Maryam Mirrajei

**Affiliations:** 1 Zanjan Metabolic Disease Research Center, Department of Pediatrics, Zanjan University of Medical Sciences, Zanjan, Iran; 2 Zanjan Social Determinants of Health Research Center, Department of Pediatrics, Zanjan University of Medical Sciences, Zanjan, Iran; 3 Department of Pediatrics, Ayatollah Moussavi Hospital, Zanjan, Iran; 4 Department of Epidemiology and Biostatistics, School of Medicine, Zanjan University of Medical Sciences, Zanjan, Iran; 5 Department of Pediatrics, School of Medicine, Ayatollah Moussavi Hospital, Zanjan University of Medical Sciences, Zanjan, Iran

**Keywords:** Neonatal Intensive Care Unit (NICU), Outcome, Premature neonates, Urinary Uric acid to creatinine ratio (UUA/Cr ratio)

## Abstract

**Background:**

Perinatal asphyxia is one of the main causes of preterm infant mortality. Some studies have shown that The Urinary Uric Acid / Cr (UUA/Cr) ratio may be used as an additional marker for perinatal asphyxia.This study intend to investigate the relationship of this ratio with outcomes of preterm infants admitted to NICU.

**Methods:**

This case–control study was carried on 102 preterm newborn infants with gestational age of 30 weeks to 33 weeks and 6 days admitted in the neonatal intensive care unit.The case group, consisted of 51 premature neonates with a history of intubation, cardiopulmonary resuscitation, mechanical ventilation and Nasal continuous positive airway pressure (NCPAP) at birth, were compared with 51 matched neonates. The UUA/Cr ratio was measured in the first 24 h after birth. Complications during hospitalization, duration of hospitalization, and final outcome were evaluated.

**Results:**

The mean level of UUA/Cr ratio in case and control group were 5.4 ± 4.1 and 3.6 9 ± 2.9 respectively and this difference was statistically significant (p = 0.014). The UUA/Cr ratio were significantly higher in females, cesarean section delivery, Apgar score ≥ 8, neonates without any complication and neonates with less than 10 days of hospitalization. However, this ratio has no predictive value for the incidence of complications during hospitalization and long-term hospital stay for infants of the case group.

**Conclusions:**

The Urinary Uric Acid / Cr ratio in the first 24 h after birth in preterm neonates who underwent intubation, NCPAP or cardiopulmonary resuscitation was higher than healthy neonates.

## Introduction

Preterm infants may need various diagnostic and therapeutic interventions according to their gestational age and medical conditions. These interventions may be expensive and cause long-term hospitalization. The mortality and morbidity of preterm infants admitted to the Neonatal Intensive Care Unit (NICU) are still major concerns for pediatricians and neonatal specialists [1] Although numerous factors are involved in the final outcome of preterm infants, perinatal asphyxia is one of the main causes of preterm infant mortality in developed countries [2].

While factors such as Apgar score and arterial blood pH have been suggested as predictors of neonatal mortality in asphyxia [3], according to the American Academy of Pediatrics and the American College of Obstetricians and Gynecologists “The Apgar score alone cannot be considered to be evidence of or a consequence of asphyxia”.[4].

In recent years, some studies have shown that The UUA/Cr ratio may be used as an additional marker for perinatal asphyxia in full-term and preterm infants. Within 24 h of birth, UUA/Cr ratio is a simple, fast, and inexpensive way to diagnose hypoxic episodes of NICU admitted patients compared with other markers such as xanthine, hypoxanthine and ascorbic acid [5–9]. The Urinary Uric Acid / Cr ratio, as a biochemical marker, can support the clinical diagnosis and severity of asphyxia previously determined by the Apgar score. However, at least in a cohort study, this ratio was not a good predictor for outcomes of patients compared to some other biomarkers [10]. The aim of this study was to evaluate the Urinary Uric Acid / Cr ratio in preterm infants who have undergone aggressive resuscitation or mechanical ventilation at birth in comparison with controls. We also evaluated the course of hospitalization of these two groups, to find out if this biochemical marker may be used as a simple and inexpensive method to determine the final outcome of infants admitted to NICU.

## Material and Methods

This case–control study was carried on 102 preterm newborn infants with gestational age of 30 weeks to 33 weeks and 6 days admitted in the neonatal intensive care unit of Mousavi Hospital in Zanjan, Iran from Dec 2018 to Jan2020. The sample size was calculated by the following formula.With a statistical power of 80% and assuming an error of the first type of 0.05, total number of samples was calculated as 51 patients in case and control groups which were selected by simple randomization.

$$n = \frac{{\left[ {Z_{{1 - \frac{\alpha }{2}}} + Z_{1 - \beta } } \right]^{2} \left[ {\sigma_{1}^{2} + \sigma_{2}^{2} } \right]}}{{(\mu_{1} - \mu_{2} )^{2} }} = \frac{{(1.96 + 1.28)^{2} (.25 + 4)}}{{(3.3 - 1.4)^{2} }} = 51$$

The case group consisted of premature neonates with a history of intubation, cardiopulmonary resuscitation, mechanical ventilation and Nasal continuous positive airway pressure (NCPAP) at birth. The controls were 51 matched premature neonates, admitted in NICU who did not need the mentioned procedures.

Infants with congenital malformations, suspected of having metabolic disease and congenital kidney disease, and infants born from mothers with preeclampsia, using drugs with respiratory depression, hypertension, and diabetes were not included in the study.

In all neonates participating in the study, 5 cc random urine sample was taken by a neonatal urinary bag and was stored in the refrigerator until the analysis. The Urinary Uric Acid / Cr ratio was determined in less than 24 h in the laboratory of Mousavi Hospital in Zanjan by auto analyzer Biolis 50 i, Prestige, Japan by spectrophotometric uricase method. All information about the course of hospitalization of infants until the last day of hospitalization as well as the Urinary Uric Acid / Cr ratio were recorded in the prepared questionnaire. The final outcomes defined as death of the patient, complete recovery without complications and recovery with the occurrence of special complications were also recorded and the type of complication was specified.

The occurrence of sepsis, intracranial hemorrhage, shock, respiratory failure, seizures, necrotizing enterocolitis, cardiovascular problems, renal vein thrombosis and kidney failure were considered as complications.

Data were analyzed by SPSS software version 16. Qualitative data in the form of tables and Chart were analyzed by Chi-square test. Quantitative data were expressed by mean and standard deviation and analyzed by t-test and Mann_ Whitney tests. A significant level for all analyzes was considered 0.05. This project was approved by Ethics Committee of Zanjan University of medical sciences (Ethics ID: IR.ZUMS.REC.1398.188). All methods were carried out in accordance with relevant guidelines and regulations and an informed consent was obtained from a parent and/or legal guardian.

## Results

One hundred two newborn preterm infants, 45 females (44.1%) and 57males (55.9%), hospitalized in NICU of Mousavi hospital, were enrolled. The demographic data and study variables are shown in Table 1. There was no significant difference between the two groups in terms of gender (p = 0.844). The mean weight in the case and control groups were1608 and 1806 g respectively. The difference between cases and controls in the weight group of 1000 to 1499 g and 1500 to 1999 g were not significant ( p value = 0 0.134 and p value = 0 0.695 respectively).

**Table 1:** The characteristic data of study variables

variables	total	case	control	P value
Gender				
Female	45(44.1%)	23	22	0.844
Male	57(55.9%)	28	29	
Type of delivery				
NVD	15(14.7%)	2	13	0.002
C/S	87(85.3%)	49	38	
Birth weight(gram)				
1499–1000	30(29.4%)	19	11	0.002
1999–1500	55(53.9%)	28	27	
≥ 2000	17(16.7%)	4	13	
5^th^ minute Apgar score				
< 8	13(12.7%)	11	2	0.007
≥ 8	89(87.3%)	40	49	
complications				
+	5(4.9%)	5	0	0.022
-	97(95.1%)	46	51	
Days of Hospitalization				
< 10	38(38.2%)	11	28	0.01
≥ 10	64(61.8%)	40	23	

Five patients in the case group showed complications but the controls did not show any complication and the difference between the two groups was significant (p value = 0.022). In the case group, 33 (64.7%) infants received surfactant and the most common morbidity during hospitalization were sepsis and germinal matrix hemorrhage (GMH). Sixty four patients (61.8%) were hospitalized more than 10 days in both groups and the number of hospitalization days differed significantly between cases and controls (p value = 0.01). The 5^th^ minute Apgar scores were less than 8 in 13 neonates (12.7%) and there was a significant difference between case and control groups regarding the 5^th^ minute Apgar scores (p value = 0 0.007).

The mean urine level of uric acid in the case group was 57.4 ± 64 mg/dl and among the controls was 50.7 ± 34.9 mg/dl and this difference was not statistically significant (p = 0.521). The mean urine creatinine level was 13.6 ± 10.8 mg/dl in cases and 19.6 ± 16.9 mg/dl in control group and this difference was statistically significant (p = 0.035). The mean level of UUA/Cr ratio was 5.4 ± 4.1 in case neonates and 3.6 9 ± 2.9 in control neonates and this difference was statistically significant (p = 0.014).

The UUA/Cr ratio according to gender, type of delivery, birth weight, Apgar score, complications and days of hospitalization are shown in the Tables 2 and 3.The difference of UUA/Cr ratio between cases and controls were significant in females (p = 0.017), cesarean section delivery (p = 0.016), 5^th^ minute Apgar scores ≥ 8 (p = 0.007), neonates without complication (p = 0.048) and neonates with less than 10 days of hospitalization (p = 0.039).

**Table 2:** The Urinary Uric Acid / Cr ratio according to study variables

variables	case	control	P value
Gender			
female	5.4 ± 4.0	3.1 ± 1.7	0.017
Male	5.5 ± 4.3	4.1 ± 3.6	0.190
Type of delivery			
NVD	3.4 ± 0.2	4.0 ± 2.9	0.785
C/S	5.5 ± 4.2	3.3 ± 2.9	0.016
Birth weight(gram)			
1499–1000	4.6 ± 1.8	4.6 ± 2.9	0.958
1999–1500	6.2 ± 5.2	3.9 ± 3.3	0.058
≥ 2000	3.7 ± 1.6	2.2 ± 1.2	0.59
5^th^ minute Apgar score			
≥ 8	5.8 ± 4.4	3.6 ± 3.3	0.007
< 8	4.1 ± 2.4	4.4 ± 1.9	0.885
Complications			
+	8.7 ± 3.9	-	-
-	5.1 ± 4.1	3.7 ± 3.0	0.048
Days of Hospitalization			
< 10	4.9 ± 4.6	2.8 ± 1.8	0.039
≥ 10	5.7 ± 4.0	5.0 ± 3.9	0.576

**Table 3:** Comparison of the means of uric acid, creatinine and uric acid to creatinine ratio in the two groups according to effective variables

variables		Uric Acid			Creatinin			UUA/Cr ratio		
		case	control	P-value	case	control	P-value	case	control	P-value
Gender	female	48.8±7.9	50.1±9.6	0.89	13.3±2.2	17.3±2.2	0.26	5.4±0.8	3.1±0.4	0.017
	male	64±15.2	50.9±4.7	0.39	13.9±2.1	21.4±3.6	.006	5.5±0.8	4.1±0.7	0.191
Type of delivery	NVD	24.7±4.3	59.9±9.5	0.18	7.1±.95	18.8±3.3	.005	3.4±0.1	4.0±0.8	0.48
	C/S	58.7±9.4	47.6±5.7	.35	13.8±1.6	19.9±3.0	.061	5.6±0.6	3.6±0.5	.012
Birth weight(gram)	1000-1499	67.7±21.5	52.9±8.2	.61	13.0±2.7	12.7±1.9	0.92	4.6±0.4	4.5±0.8	0.918
	1500-1999	47.8±7.8	51.4±8.1	.75	12.7±1.9	21.1±4.2	0.08	6.3±.98	4.0±.67	0.063
	≥2000	75.7±7.1	47.4±7.7	.024	22.5±4.3	23.3±2.8	.895	3.7±0.8	2.3±.33	0.06
5^th^ minute Apgar score	<8	58.7±10.9	50.4±5.0	.466	13.3±1.7	19.9±2.4	.038	5.8±0.7	3.7±.43	0.008
	≥8	52.6±14.6	58.3±29.9	.88	14.5±3.5	12.1±2.8	.786	4.2±.74	4.5±1.4	.874
Complications	+	47.5±15.9	---	---	7.3±2.8	----	----	8.8±1.7	----	----
	-	58.5±9.9	50.1±4.9	.47	14.3±1.6	19.6±2.4	.07	5.1±.6	3.7±0.4	.048
Days of Hospitalization	<10	49.4±8.7	44.8±4.9	0.62	15.8±2.8	21.9±3.2	0.16	4.9±1.2	2.9±.32	.039
	≥10	60.4±12.1	60.9±10.0	.98	12.8±1.8	15.8±3.3	0.47	5.7±.70	5.1±0.9	0.58

**Fig. 1:**
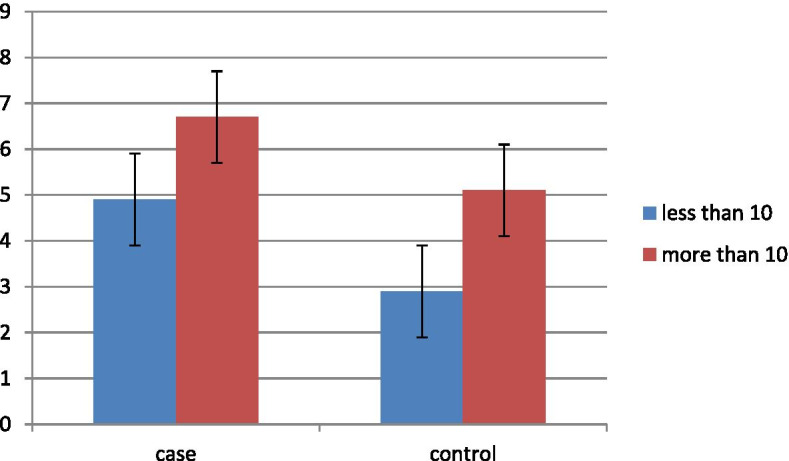
Comparison of the mean of uric acid to creatinine ratio in case and control groups according to length of hospital stay

In order to evaluate the predictive value of UUA/Cr ratio for the duration of hospitalization, the following results were obtained: In case group, this ratio in neonates with a hospital stay of ≥ 10 days was 5.7 ± 4.0 compared to 4.9 ± 4.6 in neonates with hospital stay of less than 10 days. The difference of hospital stay in case group was not significant (p = 0.840). But in the control group this ratio in neonates with a hospital stay of ≥ 10 days was 5.0 ± 3.9 compared to 2.8 ± 1.8 in neonates with hospital stay of less than 10 days. This difference is statistically significant (p = 0.040). The mean and standard deviation of UUA/Cr ratio in the case group, was not significantly different between neonates with and without complications (p = 0.064). Figure 1 shows the Comparison of the mean of uric acid to creatinine ratio in case and control groups according to length of hospital stay.

It is noteworthy that no mortality was observed in any of the case and control groups and all neonates were discharged in good condition after hospitalization, so it was not possible to evaluate the predicting value of UUA/Cr ratio in neonatal mortality.

## Discussion

In our study, the mean level of UUA/Cr ratio in cases were significantly higher than control group 5.4 ± 4.1 and 3.6 9 ± 2.9 respectively (p = 0.014). The UUA/Cr ratio were significantly higher in cases compared to controls in females, cesarean section delivery, Apgar score ≥ 8, neonates without any complication and neonates with less than 10 days of hospitalization.

Hypoxia disrupts the brain's oxidative metabolism, leading to anaerobic glycolysis for producing ATP. This causes lesser ATP production and accumulation of AMP and ADP. As a result of the catabolism of these substances, Inosine and hypoxanthine are formed which are converted to uric acid by hypoxanthine oxidase. The combination of these events and impaired renal function leads to increased blood uric acid and its secretion in the urine [2, 11, 12]. Other tests such as blood PH, lactate and base deficit may be used in asphyxia, however, they are invasive, need special instruments and recede after resuscitation and breathing establishments [11]. Therefore, UUA/Cr ratio may be a better and an easy access marker for diagnosis of tissue hypoxia [12].

Our study showed a significant higher ratio of UUA/Cr in premature neonates admitted in NICU suffering hypoxia. Several other studies have reached similar results [2, 3, 6, 8, 9, 13–21]. In the case –control study of Sreekrishna et al. on 50 normal neonates and 50 newborn infant with asphyxia, the mean Urinary Uric Acid / Cr ratio in case and control group was 2.8 ± 0.9 and 0.8 ± 0.2 respectively which shows a significant higher ratio in case group (P < 0.001) [2].

Some studies have also used Uric Acid / Cr ratio to determine the patient’s outcomes. In the study of Nariman et al. on 362 preterm infants with a mean gestational age of 32.7 ± 3.9 weeks, the mean of UUA/Cr ratio in case group was significantly higher than normal neonates (3.30 ± 1.95 vs. 1.36 ± 0.42. P = 0.0001). They concluded that the UUA/Cr ratio may be a noninvasive and inexpensive marker for detecting the outcome of ill neonate admitted in NICU [7]. However, in the cohort study of Tekgul et al. 21 newborn infants with asphyxia were followed for 2 years. No statistically significant difference in UUA/Cr ratios in patients with moderate to severe hypoxic-ischemic encephalopathy was found compared to patients with mild hypoxic-ischemic encephalopathy (3.60 vs. 1.85). They stated that compared to other biomarkers like IL-6 levels in cerebrospinal fluid, this ratio was not a good predictor of outcomes in patients [10].

Bellos et al. in a review article on 14 studies evaluated the UUA/Cr ratio in neonates with asphyxia and its role in prediction of patients’ outcome. They found that UUA/Cr ratio was significantly higher in case group with perinatal asphyxia than in normal control group. They concluded that this ratio is a simple and fast biomarker to determine neonatal asphyxia but its role in predicting long term neurologic outcome and patients’ mortality should be more investigated [22].

The correlation between the severity of asphyxia and UUA/Cr ratio has been studied by Bader et al. on 18 newborns with asphyxia and 50 healthy newborn infants. The mean UUA/Cr ratio in the case group was significantly higher than control group (2.06 ± 1.12, vs. 0.64 ± 0.48; P < 0.001). A significant correlation was shown between this ratio and severity of asphyxia (r = 0.86, P < 0.01) [23].

Akisü et al. reached the same results. They found a correlation between the UUA/Cr ratio and the severity of the hypoxic-ischemic encephalopathy (r = 0.84; P < 0.001) [12].

In our study, the UUA/Cr ratio was significantly higher in cases in females, but the study of Khalesi did not show any relation regarding to gender [8]. The greater UUA/Cr ratio in females may be due to the fact that the urinary creatinine of females is less than males [24], therefore it may be expected that the increase of urinary uric acid ratio in these patients can be better identified.

We found a statistically significant relationship between 5^th^ minute Apgar scores < 8 and UUA/Cr ratio in the case group. This can be expected because when the Apgar score is above 8, the anaerobic glycolysis process, which leads to increased urinary excretion of uric acid, is not active. Several studies found a negative correlation between 5^th^ minute Apgar score and UUA/Cr ratio [2, 7, 14].

Among neonates without complications, UUA/Cr ratio was significantly higher in case group compared to controls. This is expected because cases may suffer more hypoxia than controls. It is also expected that this ratio in the case group with complications is even higher than the control group, but due to the fact that no complication was observed in the control group, this comparison was not possible.

In neonates who were hospitalized for less than 10 days, UUA/Cr ratio was higher in the case group compared to control group. But this finding was not observed in hospitalization for more than 10 days. In our opinion, in the control group, neonates with more than 10 days of hospitalization, may probably have some unforeseen problems which increased this ratio, so that they did not show any difference with the case group. On the other hand, muscle mass of patients with more than 10 days of hospitalization may be decreased for various reasons. These factors include underlying disease, nutrition, etc. Decreased muscle mass will reduce urinary creatinine excretion and increase this ratio.

## Conclusion

The UUA/Cr ratio in the first 24 h after birth in preterm infants (30 to 33 weeks and 6 days) who underwent intubation, NCPAP or cardiopulmonary resuscitation was significantly higher than healthy premature neonates. We may hypothesize that UUA/Cr ratio can be a good predictor of increasing the length of hospital stay in healthy preterm infants. However, this ratio has no predictive value for the incidence of complications during hospitalization or long-term hospitalization stay in sick premature newborns.

In order to achieve the predictive role of this ratio for the long-term outcome of patients, it is recommended that cohort studies be designed and implemented in this regard.

## Data Availability

All data and materials are available and potentially shareable on request. The in charge person is Dr Parisa Khoshnevisasl. The phone number is + 989,122,429,374 and the email is khoshnevis@zums.ac.ir.

## References

[1] Sadeghzadeh M, Khoshnevisasl P, Parvaneh M, Mousavinasab NJIjocn. Early and Late Outcome of Premature Newborns with History of Neonatal Intensive Care Units Admission at 6 Years Old in Zanjan, Northwestern Iran. Iran J Child Neurol. 2016;10(2):67. PMC4885157 27247586

[2] Sreekrishna YE, HL SA. Study of urinary uric acid to creatinine ratio as a biochemical marker of perinatal asphyxia and its correlation with Apgar Score. Int J Contemp Pediatrics. 2018;5(4):1485.

[3] Patel KP, Makadia MG, Patel VI, Nilayangode HN, Nimbalkar SMJJoc. Urinary uric acid/creatinine ratio-a marker for perinatal asphyxia. J Clin Diagnostic Res. 2017;11(1):SC08. 10.7860/JCDR/2017/22697.9267 PMC5324459 28274014

[4] Committee Opinion No (2015). 644: The Apgar Score. Obstetrics Gynecol.

[5] Chen H-J, Yau K-IT, Tsai K-S. Urinary uric acid/creatinine ratio as an additional marker of perinatal asphyxia. J Formosan Med Assoc. 2000;99(10):771–4. 11061072

[6] Krishnan AR, Nayak P, Bellipady SS, Shenoy RD. Urine uric acid creatinine ratio as a diagnostic and prognostic marker of neonatal birth asphyxia. Asian J Clin Pediatrics Neonatol. 2020;8(2):6–10. 10.47009/ajcpn.2020.8.2.2.

[7] Nariman S, Mosayebi Z, Sagheb S, Rastad H, Hosseininodeh S S. Urinary uric acid/creatinine ratio as a marker of mortality and unfavorable outcome in NICU-admitted neonates. Iran J Pediatr. 2016;26(4):e5739. 10.5812/ijp.5739 PMC5046843 27729961

[8] khalesi N, kosravi N, saiedi V, kalani MJRJoMS. comparison of uric acid to creatinine ratio in term infants with perinatal asphyxia and healthy neonates admitted in Ali-Asghar and Akbar-Abadi hospitals during 2010–2011. Razi J Med Sci. 2014;21(125):119–25.

[9] Kumar D, Chaudhari PK, Chaudhary A, Kamal S (2016). Urinary uric acid and creatinine ratio as a marker of perinatal asphyxia. IOSR J Dent Med Sci.

[10] Tekgul H, Yalaz M, Kutukculer N, Ozbek S, Kose T, Akisu M (2004). Value of biochemical markers for outcome in term infants with asphyxia. Pediatric Neurol.

[11] Gubbala T, G. N. SK. Study of urinary uric acid and creatinine ratio as a marker for perinatal asphyxia. Int J Contemp Pediatrics. 2020;7(5):4

[12] AKISÜ M, KÜLTÜRSAY NJPI. Value of the urinary uric acid to creatinine ratio in term infants with perinatal asphyxia. Pediatrics Int. 1998;40(1):78–81. 10.1111/j.1442-200x.1998.tb01408.x 9583207

[13] Bhongir AV, Yakama AVV, Saha S, Radia SB, Pabbati JJEjp. The urinary uric acid/creatinine ratio is an adjuvant marker for perinatal asphyxia. Eur J Pharm Med Res. 2015;2(5):520. PMC4793967 26998526

[14] Basu P, Som S, Choudhuri N, Das HJIjocb. Correlation between Apgar score and urinary uric acid to creatinine ratio in perinatal asphyxia. Indian J Clin Biochem. 2008;23(4):361–4. 10.1007/s12291-008-0079-2 PMC3453141 23105787

[15] Banupriya C, Ratnakar, Doureradjou P, Mondal N, Vishnu B, Koner BC. Can urinary excretion rate of malondialdehyde, uric acid and protein predict the severity and impending death in perinatal asphyxia? Clin Biochem. 2008;41(12):968–73. 10.1016/j.clinbiochem.2008.04.011 18471999

[16] Sharma I, Verma G, Sharma N, Sankhwar D (2020). Utility of APGAR score, urinary uric acid and creatinine ratio with perinatal asphyxia. IAIM.

[17] Wadhwa V, Popli V, Kandukoori PK, Kansal P (2018). The Role of Urinary Uric Acid to Cretonne Ratio as a Marker of Prenatal Asphyxia and Its Severity in Newborns. IOSR J Dental Med Sci.

[18] Suman KV, Beeregowda Y, Shashidhar K (2019). Correlation of Urinary Uric Acid, Creatinine Ratio with the severity of Hypoxic Ischemic Encephalopathy. J Adv Med Dental Sci Res.

[19] Choudhary L, Palsania S, Berwal P, Sauparna C, Maheshwari A (2017). Study of urinary uric acid and creatinine ratio as a marker of perinatal asphyxia and its correlation with different stages of hypoxic ischemic encephalopathy. J Pregnancy Child Health.

[20] Krishnana EPV, Sekar SP (2017). Study of urinary uric acid and creatinine ratio as a marker of neonatal asphyxia for babies born in a tertiary care hospital. IJRMS.

[21] Saranya SJIIJoMP. Study of urinary uric acid and creatinine ratio as a marker for neonatal asphyxia. IP Int J Med Paediatrics Oncol. 2019;5(2):66–8.

[22] Bellos I, Fitrou G, Pergialiotis V, Perrea DN, Papantoniou N, Daskalakis GJTJoM-F. Random urine uric acid to creatinine and prediction of perinatal asphyxia: a meta-analysis. J Matern Fetal Neonatal Med. 2019;32(22):3864–70. 10.1080/14767058.2018.1471677 29712490

[23] Bader D, Gozal D, Weinger-Abend M, Berger A, Lanir A (1995). Neonatal urinary uric acid/ceratinine ratio as an additional marker of perinatal asphyxia. Eur J Pediatrics.

[24] Kwon S-Y, Na Y-AJKJCLS. Concentration of serum and urine creatinine in children and adolescents. Korean J Clin Lab Sci. 2014;46(4):117–23.

